# Supplementary material for: Pressure-Tuning Superconductivity in Noncentrosymmetric Topological Materials ZrRuAs
Source: Materials (Basel). 2022 Nov 1;15(21):7694. doi: 10.3390/ma15217694 (PMC9659151; doi:10.3390/ma15217694)
Supplement: Supplementary file 1 [file materials-15-07694-s001.zip › materials-1972543-supplementary-done.pdf]

materials

Supplementary Materials

Pressure-Tuning Superconductivity in Noncentrosymmetric Topological Materials ZrRuAs

Table S1. The relaxed lattice structure of ZrRuAs at different pressures. The first column shows the theoretically determined pressures at which experiments were also conducted. The relaxed lattice constants, the atomic positions of Zr and Ru are shown in the second, third, fourth, and last columns, respectively.

Pressure (GPa)	*a* (Å)	*c* (Å)	Zr	Ru
0.3	6.609	3.943	0.582, 0.000, 0.000	0.244 0.000 0.500
0.9	6.603	3.939	0.582, 0.000, 0.000	0.244 0.000 0.500
2.1	6.589	3.930	0.582, 0.000, 0.000	0.244 0.000 0.500
6.0	6.543	3.911	0.581, 0.000, 0.000	0.244 0.000 0.500
13.5	6.476	3.867	0.581, 0.000, 0.000	0.245 0.000 0.500
17.1	6.447	3.848	0.581, 0.000, 0.000	0.245 0.000 0.500
24.0	6.394	3.814	0.581, 0.000, 0.000	0.245 0.000 0.500
29.8	6.356	3.787	0.581, 0.000, 0.000	0.245 0.000 0.500

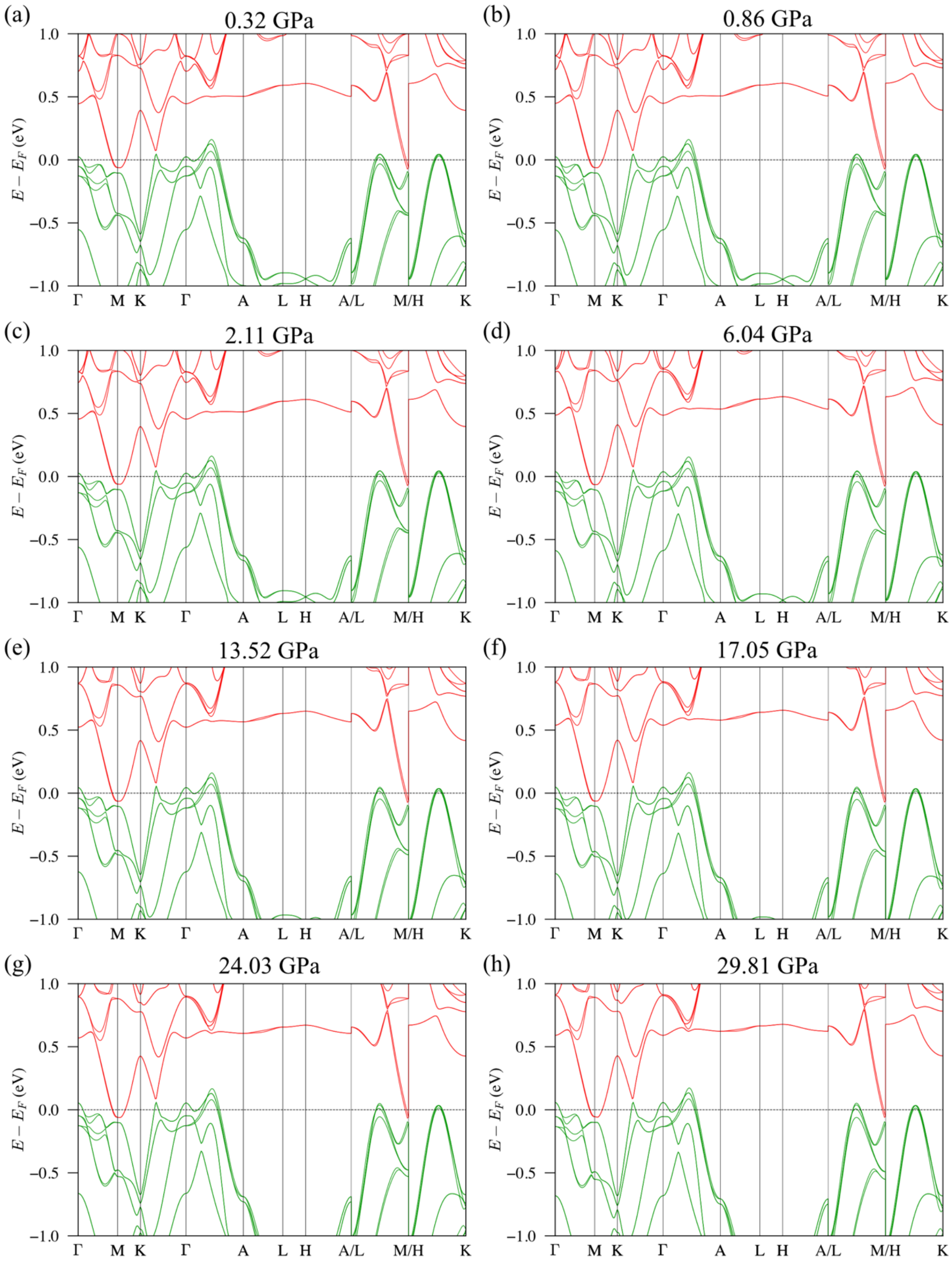

Figure S1. Electronic band structures of ZrRuAs with SOC under different pressures. Red and green lines are the conduction and valence bands, respectively.

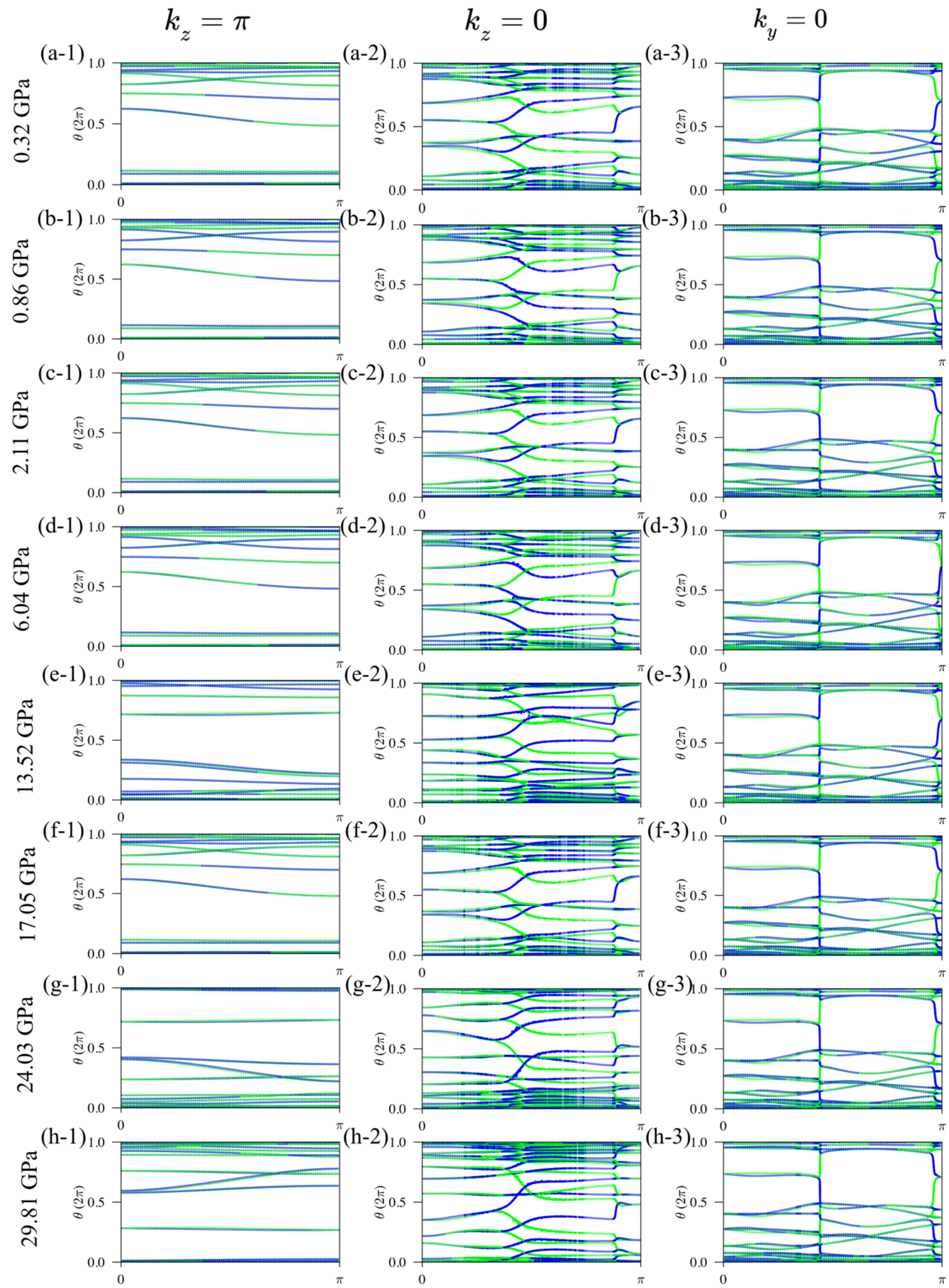

Figure S2. Wilson loops on mirror symmetric planes of ZrRuAs with SOC under different pressures. Blue and green lines denote the flows of Wannier charge centers for states in subspaces of mirror +*i* and -*i* eigenvalues, respectively. The first, second and third columns correspond to the Wilson loops on $k_z = \pi$, 0 and $k_y = 0$ planes, respectively. The values on the leftmost of each row are the pressures under which Wannier charge centers are calculated.
